# Comparing RNA extraction protocols from formalin-fixed paraffin-embedded microcore samples

**DOI:** 10.1371/journal.pone.0338439

**Published:** 2025-12-08

**Authors:** Maxime Golias, Zuzana Krupova, Pierre Defrenaix, Marie-Françoise Heymann, Dominique Heymann

**Affiliations:** 1 Excilone, Elancourt, France; 2 Nantes Université, CNRS, UMR6286, US2B, Nantes, France; 3 Institut de Cancérologie de l’Ouest, Tumor Heterogeneity and Precision Medicine Laboratory, Saint-Herblain, France; 4 Université of Sheffield, School of Medicine and Population Health, Sheffield, United Kingdom; Tehran University of Medical Sciences, IRAN, ISLAMIC REPUBLIC OF

## Abstract

The ability to analyze intratumoral heterogeneity is of great interest for both diagnostic and basic research purposes. However, currently available dissection techniques are unsuitable for routine use and hard to access financially. Recently, a novel microcore-based dissection technique has been developed by the company Excilone for studying tissue heterogeneity in formalin-fixed paraffin-embedded (FFPE) microcore samples. The use of FFPE biological samples for transcriptomic studies, coupled with their small size, remains a real barrier to dissection applications. The efficacy of five commercially available RNA extraction kits were analyzed on microcores collected from human and mice FFPE tissues. Thirty microcore samples of healthy tissue (human uterus and stomach, and murine liver and spleen) were collected and distributed equally and randomly to the five kits assessed. Microcores were collected directly from paraffin blocks using 200 µm inner diameter needles with a sample depth, variable regarding to tissue type, ranging from 450 to 600 µm. Overall RNA yield and RNA fragmentation were evaluated, and RT-qPCR analyses were carried out after deparaffinization and compared to non-deparaffinization protocols. RNA yields and RNA fragmentation varied considerably between kits and FFPE tissues analyzed. Although the main limitation of this technique is the small initial sample size, differences in qPCR efficiency were also observed. Interestingly, no significant differences were observed between deparaffinized and non-deparaffinized microcore samples. Ultimately, we demonstrate the feasibility of using FFPE microcore samples for sensitive molecular biology applications, both with and without deparaffinization. The importance of setting up an optimized workflow was emphasized by significant differences observed in outcomes of the different protocols.

## Introduction

The high degree of heterogeneity found within tumors can make establishing an accurate diagnosis a difficult task [1,2]. Microdissection techniques, which enable reliable and reproducible isolation of specific tissue areas, have been developed to address this challenge. However, these technologies are not easily accessible and integrating them into daily practice can be challenging [3,4]. Additionally, as microbiopsy techniques become more widely used, pathologists are increasingly required to make diagnoses based on limited tissue samples. This necessitates the optimization of techniques involved in comprehensive diagnosis. Therefore, there is an interest in developing techniques and protocols that enable routine implementation of microdissection.

Pathologist diagnosis is often performed on formalin-fixed paraffin-embedded tissue (FFPE). This fixation process allows biological materials to be preserved at room temperature without morphological degradation and allows analysis years after embedding. These samples, of which it is estimated that between 400 million [5] and 1 billion [6] samples are stored worldwide, represent a considerable source of information. Although long-term preservation of tissues is possible with this process, it can introduce numerous side effects that may affect the performance of downstream molecular biology techniques. Preanalytical factors, such as tissue size, formalin composition, fixation time, pH, storage conditions and duration of conservation affect the overall quality of these fixed tissues [7,8]. In addition, formalin induces protein-protein and protein-nucleic acid crosslinks [9–11] that lead to significant nucleic acid fragmentation during crosslinking dissociation [8] and suboptimal performance of extracted nucleic acids in downstream analysis compared to other preservation techniques [12]. Therefore, all extraction and purification steps must be optimized for subsequent applications. For that, dedicated protocols have been established in most molecular biology applications to reduce the impact of the FFPE process [13].

Recent developments in microdissection technologies based on 200 µm microcores, such as the MiniPunch® (Excilone) [14] and the TMA Molmed (3D Histech) [15], reflect the interests of researchers, but also pathologists, in addressing tumor heterogeneity in daily practice. These innovative sampling techniques allow precise selection of areas of interest for the study of intratumoral heterogeneity while reducing the use of available biological material. However, these microcores are limited by the small amount of initial material, therefore each step of the process requires careful optimization. Numerous studies have investigated the impact of RNA extraction protocols on subsequent applications [16–23]. However, these comparative studies utilized whole tissue sections or larger cores where key parameters such as surface area to volume and paraffin content differ. This constitutes a critical methodological gap as direct applicability of existing kit comparisons to microcores remains uncertain.

To address this uncertainty, we compared five commercial FFPE RNA extraction kits using microcore samples taken from FFPE blocks (four different tissues) by using a dedicated needle commercialized by Excilone with a 200 µm internal diameter. As microcore samples were collected from tissue-enriched areas, the amount of paraffin associated with was limited. We then compared two extraction methods for each kit, with or without a deparaffinization step. Reducing the number of steps shortens extraction protocol time and reduces the risk of microcore loss during each step. To determine the most suitable protocol for sampling of FFPE microcores, various parameters, such as RNA yield, fragmentation and qPCR efficiency were analyzed.

This study demonstrates the feasibility of using non-deparaffinized FFPE microcores to analyze tissue heterogeneity across tissue types and RNA extraction protocols. Also emphasized is the importance of selecting the correct protocol for such microcore samples to maximize reliability of molecular diagnostic and research applications.

## Materials and methods

### Sample preparation

Healthy human uterus and stomach tissues were provided by the biological resource center - tumor tissue library at the Institut de Cancérologie de l’Ouest (ICO) in accordance with French legislation (Centre de Ressources Biologiques-Tumorothèque de l’ICO, Saint-Herblain, France, Declaration Number: DC-2018-3321) and written informed consent was obtained for each patient as required by French legislation and the French ethics committee (Comité de Protection des Personnes, CCP) for the protection of human rights [24]. Healthy murine liver and spleen tissues were obtained in accordance with the protocol validated by the French ethics committee of the “Pays de la Loire” (CEEA-PdL-06) and authorized by the French State Department of Agriculture and Fisheries (authorization APAFIS # 2168-2019080709313558 v6). Briefly, tissues were fixed in 10% neutral buffered formalin at room temperature. Tissues were then dehydrated and embedded in paraffin and stored at room temperature. One FFPE block from each tissue was used for this study.

### FFPE microcores’ collection

Microcore samples were collected using the Manual Tissue Arrayer MTA-1 (Alpha Metrix) with a 200 µm internal diameter needle attached. Coring was performed by punching directly into the donor block. Microcores from the stomach were obtained from the fundic mucosa, whereas uterine microcore samples were located from the myometrium. Murine microcore samples were collected from hepatic and splenic parenchyma. Collected microcore samples were randomly distributed. Five microcore samples of each tissue were distributed to each kit assessed. Following the sampling step, microcore sample location was validated with HE staining of the donor block. Length and width were measured using the PathScanView software (Excilone) to allow standardization and ensure conformity of the collected microcore samples.

### Nucleic acid extraction

Five commercial kits were compared. Nucleospin^®^ totalRNA FFPE XS (Macherey-Nagel; Nucleospin, batch number 2303.002), Quick-RNA^TM^ FFPE MiniPrep (Zymo Research; Quick-RNA, batch number 223804), PureLink^TM^ FFPE Total RNA isolation kit (Invitrogen, Thermo Fisher Scientific; PureLink^TM^, batch number 2592172), RNeasy^®^ FFPE Kit, (Qiagen; RNeasy, batch number 175023581) and truxTRAC^TM^ FFPE RNA microtube Kit-Column Purification (Covaris; truXTRAC, batch number 28608851) were used in this study. Non-deparaffinized microcore samples did not undergo the prior deparaffinization step. A 4 h incubation time with proteinase K was performed to allow for complete microcore sample dissociation. DNase1 treatment was performed systematically to reduce risk of gDNA contamination. Two successive elutions were carried out. The first elution was performed with the minimum volume recommended by the supplier. A second elution was collected in a separate tube using additional volume required to a maximum recommended volume. Total RNA yield of the eluates was measured. RNA integrity assessment and qPCR analysis were performed using the first elution containing higher RNA concentration. All other procedures were performed according to the manufacturer’s instructions. Extracted RNA was stored at −80°C.

### Nucleic acid quantification and integrity assessment

The RNA concentration was measured using the Qubit^TM^ 4.0 Fluorometer (Invitrogen, Thermo Fisher Scientific). The Qubit^TM^ RNA High Sensitivity Assay kit (Invitrogen, Thermo Fisher Scientific) was used for low input samples. RNA integrity was assessed via capillary chip electrophoresis. Chips were used with the RNA 6000 Pico kit (Agilent) and run on the Agilent 2100 BioAnalyzer. The same amount of total RNA was used for all conditions derived from the same tissue.

### RT-qPCR

Reverse transcription of RNA to cDNA was performed using the OneScript RT Mix with the g/DNA out kit (Ozyme) on the SimpliAmp Thermal Cycler (Applied biosystems, Thermo Fisher Scientific). Depending on the initial amount of eluted RNA, between 20 and 100 ng of RNA were reverse transcribed per sample. To optimize the PCR reaction by reducing effects of severe RNA fragmentation, primers were designed with the objective of yielding a small amplicon size. Housekeeping genes *RPL27* and *POLR2A* were targeted in mouse tissues. Similarly, *GAPDH*, *POLR2A* and *PGK1* were targeted in human specimens. The sequences of the primers designed by NCBI Primer designing tool and synthetized by Eurofins are listed in Table 1. The *GAPDH* targeting sequences (QT00079247, Qiagen) were provided by Qiagen. The PowerUp^TM^ SYBR^®^ Green Master Mix (Applied Biosystems) was used for qPCR analysis, using the thermocycler QuantStudio^TM^ 5 Real-Time PCR System (Applied Biosystems, Thermo Fisher Scientific). After the initial denaturation step, PCR amplification included 40 cycles composed of a denaturation step at 95°C for 15s and an amplification step at 60°C for 1 min. Results were analyzed using the QuantStudio design and analysis software 2.7.0 (Applied Biosystems, Thermo Fisher Scientific).

**Table 1:** Characteristics of designed primers.

Gene	Species	Primers	Sequence	Length (pb)
*POLR2A*	Human	Forward	5’ AACTGGAGACAGCAAGGTCG 3’	47
		Reverse	5’ TCATCCGCAGCAGGTTACAG 3’	
*PGK1*	Human	Forward	5’ GCGGGTCGTTATGAGAGTCG 3’	85
		Reverse	5’ TGGGACAGCAGCCTTAATCC 3’	
*RPL27*	Mouse	Forward	5’ CCTCATGCCCACAAGGTACTC 3’	76
		Reverse	5’ TGGGTCCCTGAACACATCCT 3’	
*POLR2A*	Mouse	Forward	5’ AAGGATGTAGACCCTGTGCG 3’	69
		Reverse	5’CTCAATGCCCAGTACCGTGA 3’	

### Statistical analysis

This work is a blind study in which two independent operators performed microcore preparation and RNA extraction on randomized samples. Statistical analysis and graphical representations were performed using GraphPad Prism 8.0.2 software (GraphPad Software). All comparisons analyzed were performed by pairwise approach (1 vs 1). A Kruskal Wallis test with Dunn’s correction was performed to evaluate the difference between kits and between deparaffinized and non-deparaffinized conditions. A *p-value *≤ 0.05 was considered statistically significant.

## Results

### Yield of extracted RNA depends on kit and isolation conditions

**Fig 1:**
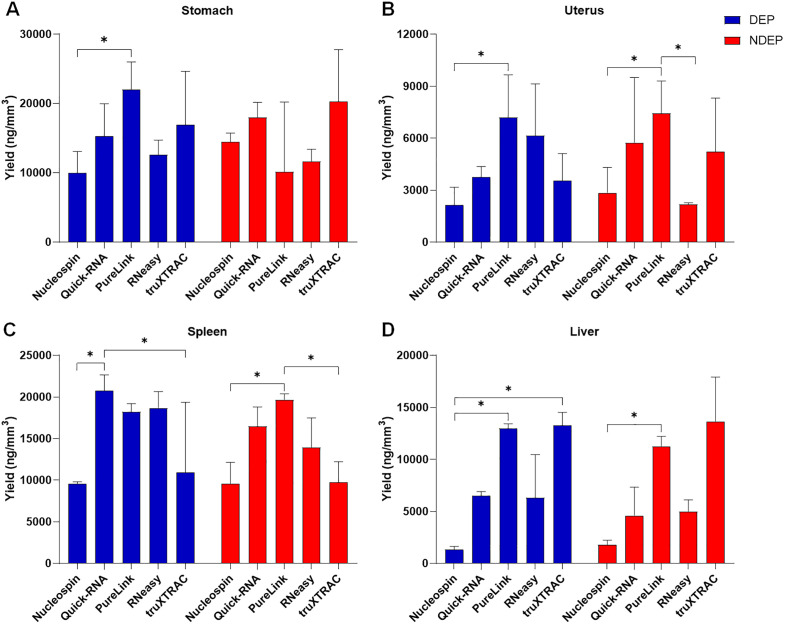
Quantitative analysis of eluted RNA obtained across kits and conditions. RNA was extracted from deparaffinized (DEP, blue bars) or non-deparaffinized (NDEP, red bars) conditions of human stomach (A), human uterus (B), mouse spleen (C) or mouse liver (D). The yields, expressed in ng/mm^3^, were normalized to the size of the associated microcore sample to correct for variations in sampling depth between microcores. n = 3, except Stomach RNeasy (NDEP) (n = 2) and Liver truXTRAC (NDEP) (n = 2). Kruskal Wallis with uncorrected Dunn’s test, *: p < 0.05.

After microcore collection and measurement, RNA was extracted and quantified (Table 2). Due to variation in microcore length (S1 Fig), and to allow kit efficiency comparison, yields of extracted RNA were normalized and expressed in ng/mm^3^ (Fig 1).

**Table 2:** Quantitative and qualitative analysis of RNA isolated using different kits and paraffin removal conditions.

Tissue	Kit	Condition	Yield (ng/mm^3^)	Concentration (ng/µL)	DV_200_ (%)	Tissue	Kit	Condition	Yield (ng/mm^3^)	Concentration (ng/µL)	DV_200_ (%)
**Stomach**	Nucleospin	DEP	9.983	6.00	53	**Spleen**	Nucleospin	DEP	9.560	5.00	86
		NDEP	14.473	8.3	66			NDEP	9.549	5.58	85
	Quick-RNA	DEP	15.247	8.40	79		Quick-RNA	DEP	20.737	10.21	84
		NDEP	17.973	10.97	57			NDEP	16.484	8.53	83
	PureLink	DEP	22.051	12.97	58		PureLink	DEP	18.220	10.41	82
		NDEP	10.154	6.33	63			NDEP	19.564	10.88	85
	RNeasy	DEP	12.565	6.98	49		RNeasy	DEP	18.760	9.43	68
		NDEP	11.655*	7.68	49			NDEP	13.927	8.04	48
	truxTRAC	DEP	16.944	9.82	55		truxTRAC	DEP	10.929	4.65	74
		NDEP	20.269	10.85	55			NDEP	9.778	4.62	78
**Tissue**	**Kit**	**Condition**	**Yield (ng/mm**^**3**^)	**Concentration** **(ng/µL)**	**DV**_**200**_ **(%)**	**Tissue**	**Kit**	**Condition**	**Yield (ng/mm**^**3**^)	**Concentration** **(ng/µL)**	**DV**_**200**_ **(%)**
**Uterus**	Nucleospin	DEP	2.163	1.32	84	**Liver**	Nucleospin	DEP	1.334	1.06	26
		NDEP	2.837	1.64	82			NDEP	1.787	1.13	38
	Quick-RNA	DEP	3.761	2.12	83		Quick-RNA	DEP	6.513	4.54	70
		NDEP	5.748	3.27	82			NDEP	4.595	2.59	60
	PureLink	DEP	7.198	4.13	80		PureLink	DEP	12.959	7.95	70
		NDEP	7.433	3.94	80			NDEP	11.249	7.49	65
	RNeasy	DEP	6.142	4.09	66		RNeasy	DEP	6.311	5.01	60
		NDEP	2.184	1.31	47			NDEP	4.977	2.76	47
	truxTRAC	DEP	3.540	2.25	71		truxTRAC	DEP	13.283	6.61	84
		NDEP	5.219	2.85	68			NDEP	13.269*	7.46	74

DV_200_: Percentage of RNA fragments that have a size between 200 and 4000 bp. DEP: Deparaffinized, NDEP: Non Deparaffinized Yield n = 3 or n = 2 (*), DV_200_: n = 1).

Working with micro-quantities of tissue is associated with low RNA yield which is often close to or below the detection limits of dedicated equipment. Despite this, all kits yielded quantifiable RNA with concentrations above 1 ng/µl (Fig 1, Table 2), confirming the fundamental feasibility of RNA extraction from microcores. Given that PCR-based techniques are generally optimized for concentrations around this threshold, our results demonstrate the applicability of microcores for a substantial range of downstream molecular biology applications. Additionally, skipping the paraffin removal step was found not to affect RNA yield (Fig 1, Table 2).

RNA extraction yields were then compared according to the tissue studied. Higher RNA yield was observed in the fundic mucosa and spleen than in the liver or uterus (Fig 1A–D, Table 2), which could be explained by differences in tissue composition. Indeed, the spleen and gastric mucosa are highly cellular tissues with elevated transcriptional activity. The uterus and liver are less cellular and more fibrous, leading to a lower yield. A significant variation in yield was also observed within the same tissue depending on the kit used. Except for the stomach and uterus non-deparaffinized protocols (Fig 1A,1B, Table 2), the Nucleospin kit isolated the least RNA under all conditions with a yield up to 10 times lower than the truXTRAC kit in the liver (deparaffinized condition) (Fig 1D, Table 2).

The results for the other kits varied depending on tissue studied. Overall, the PureLink kit performed best, producing higher and more consistent RNA yields than the other kits in most conditions (Fig 1). RNA yields obtained with the Quick-RNA and truXTRAC kits varied depending on tissue analyzed. The Quick-RNA kit showed the highest yield in the spleen under deparaffinized conditions (Fig 1C), but also the lowest efficiency under certain conditions, such as for the liver under both deparaffinized and undeparaffinized conditions (Fig 1D). The same pattern was observed with the truXTRAC kit. This protocol provided the highest yield for both liver conditions (Fig 1A), as well as a yield equivalent to that of the Nucleospin kit for the spleen (Fig 1D).

### Fragmentation of RNA extracted varies between kits

Fragmentation of eluted RNA was assessed to determine which kit best preserves RNA integrity. The DV_200_ quality metric, which has been adapted specifically for highly fragmented FFPE nucleic acids, was used to quantify RNA integrity. This metric is commonly used for pre-sequencing RNA quality validation and highlights the percentage of RNA fragments longer than 200 nucleotides. Visual analysis of electropherograms was also employed to categorize the kits used in this study.

**Fig 2:**
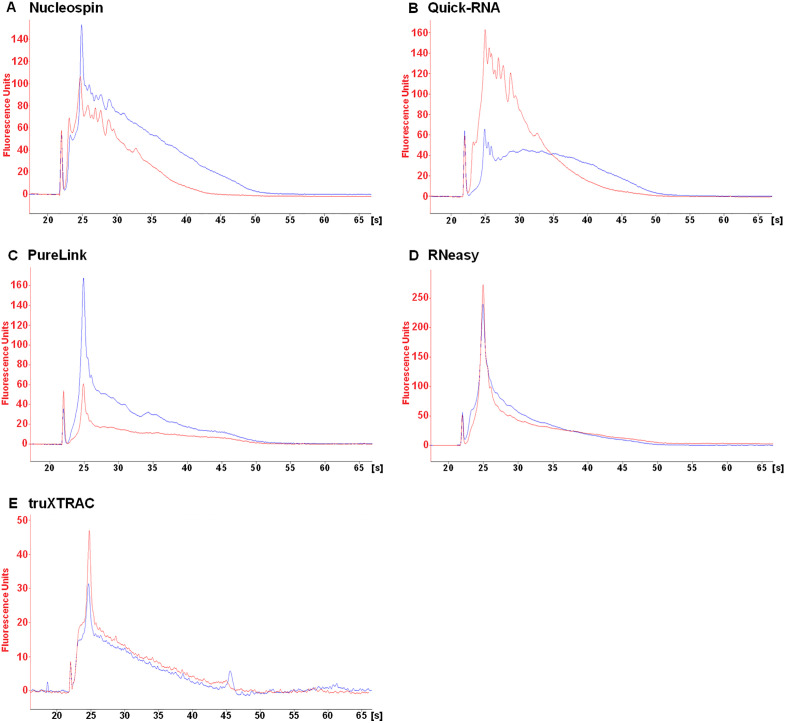
Qualitative analysis of eluted RNA from human stomach microcores. RNA extracted from deparaffinized (DEP, blue line) or non-deparaffinized (NDEP, red line) conditions was analyzed for each kit. The same amount of RNA was deposited for both conditions with each kit. Results were obtained with the RNA 6000 Pico Kit on the 2100 BioAnalyzer (Agilent). [s] migration time, in seconds.

**Fig 3:**
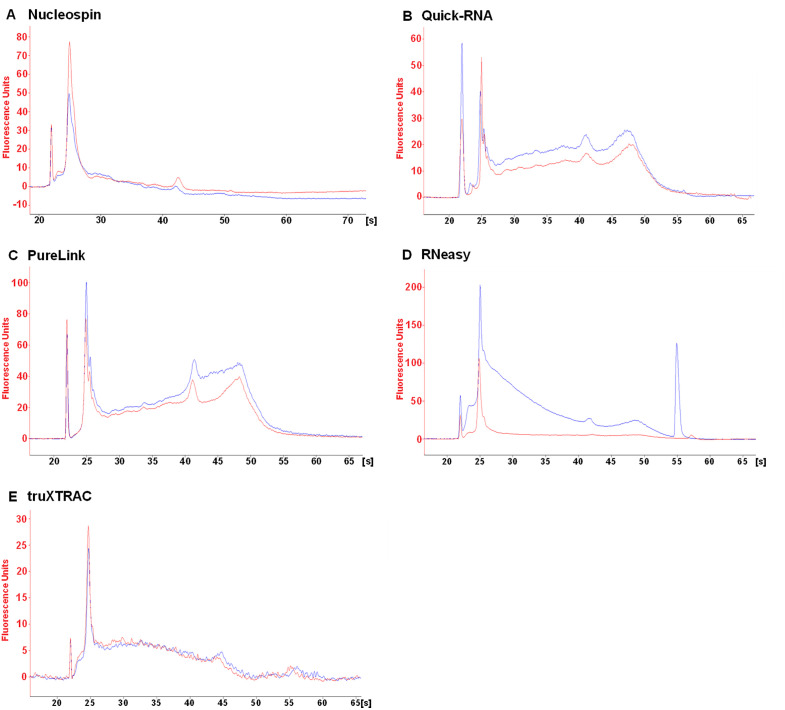
Qualitative analysis of eluted RNA from human uterus microcores. RNA extracted from deparaffinized (DEP, blue line) or non-deparaffinized (NDEP, red line) conditions was analyzed for each kit. The same amount of RNA was deposited for both conditions within each kit. Results were obtained with the RNA 6000 Pico Kit on the 2100 BioAnalyzer (Agilent). [s] migration time, in seconds.

**Fig 4:**
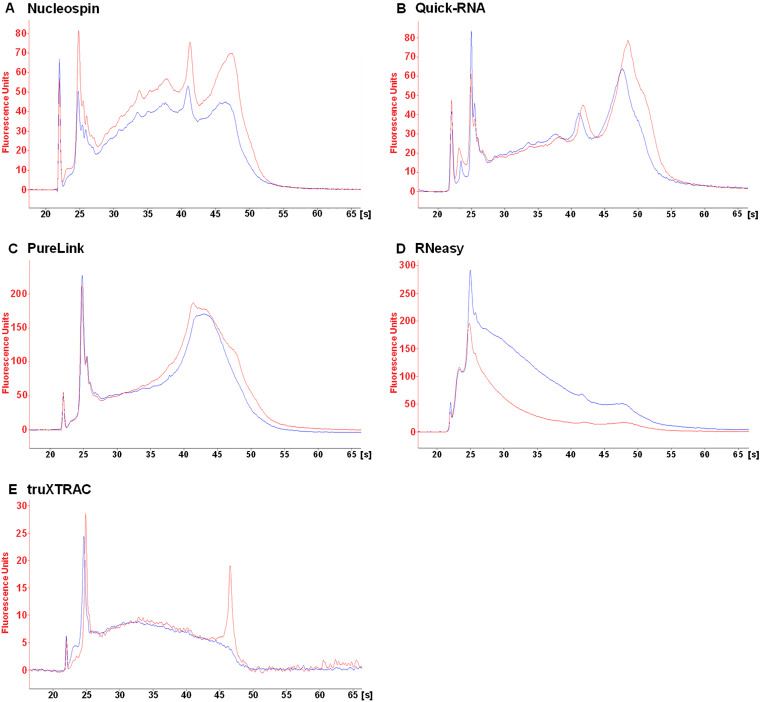
Qualitative analysis of eluted RNA from mouse spleen microcores. RNA extracted from deparaffinized (DEP, blue line) or non-deparaffinized (NDEP, red line) tissues was analyzed for each kit. The same amount of RNA was deposited for both conditions within each kit. Results were obtained with the RNA 6000 Pico Kit on the 2100 BioAnalyzer (Agilent). [s] migration time, in seconds.

**Fig 5:**
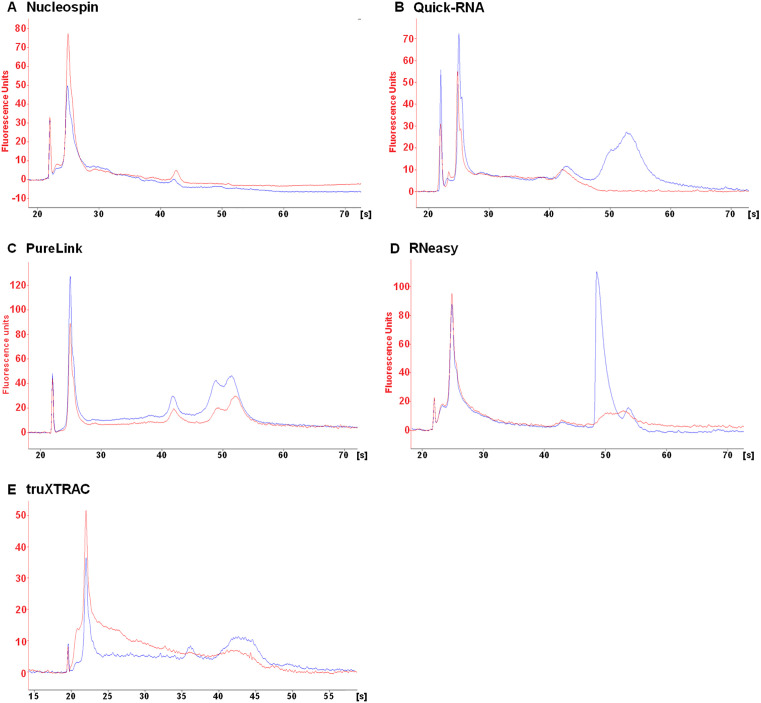
Qualitative analysis of eluted RNA from mouse liver microcores. RNA extracted from deparaffinized (DEP, blue line) or non-deparaffinized (NDEP, red line) tissue was analyzed for each kit. The same amount of RNA was deposited for both conditions within each kit. Results were obtained with the RNA 6000 Pico Kit on the 2100 BioAnalyzer (Agilent). [s] migration time, in seconds.

RNA fragmentation levels depended on the FFPE tissue block and kit used. RNAs were found to be more fragmented in the stomach (Fig 2) than in other tissues (Figs 3–5). This higher fragmentation may be due to the biological characteristics of the fundic mucosa which produces hydrochloric acid and numerous digestive enzymes, making RNA particularly susceptible to autolysis. Consequently, extensive RNA fragmentation may occur between tissue excision and fixation. The RNeasy kit led to higher levels of fragmentation. Indeed, this kit resulted in a lower DV_200_ value in both deparaffinized and non-deparaffinized conditions for three out of the four tissues analyzed (Figs 2–5, Table 2). This difference was particularly pronounced in the liver (Fig 3, Table 2) and the uterus (Fig 4, Table 2), where the DV_200_ values obtained were notably lower than those obtained with competing kits (Table 2). In addition, with the exception of the stomach, the non-deparaffinized protocol with this kit increased RNA degradation compared to the deparaffinized protocol (Figs 2–5, Table 2).

Good preservation of RNA integrity was achieved in the spleen and uterus using the Nucleospin, Quick-RNA and PureLink kits (Figs 2,3, Table 2). Furthermore, the 18S and 28S rRNAs, which are used to estimate the RNA Integrity Number, were detectable in the spleen, uterus and liver for the Quick-RNA and PureLink kits (Figs 3–5). These rRNAs were also detected in the uterus using the Nucleospin kit (Fig 4). The presence of these rRNAs confirmed the good overall preservation of RNAs under these conditions. The truXTRAC kit produced lower DV_200_ values than the three previously mentioned kits, except in the liver (Figs 2–5, Table 2), suggesting increased RNA fragmentation when using this protocol. Finally, considerable RNA fragmentation was observed in the liver using the Nucleospin kit, which resulted in a very low DV_200_ value (Fig 5, Table 2). For these four kits, the quality metric value for deparaffinized and non-deparaffinized conditions was found to be in the same range (Figs 2–5, Table 2). This finding indicates that the non-deparaffinized protocol did not increase fragmentation when using these kits. It is also important to emphasize that all quality metric values, except for one condition (Nucleospin, deparaffinized condition in liver) were above the 30% threshold generally required for RNA-seq, suggesting that the fragmentation parameter will likely not represent a limiting for such applications.

### qPCR application of microcores samples is feasible but faced limitations

The presence of contaminants associated with formalin fixation and paraffin residues can affect the reliability of subsequent experiments [7]. In addition, the degradation of biological material such as RNA impacts the outcome of experiments. Here, a functional comparison was performed to assess the applicability of our microcores for qPCR application across multiple protocols.

**Fig 6:**
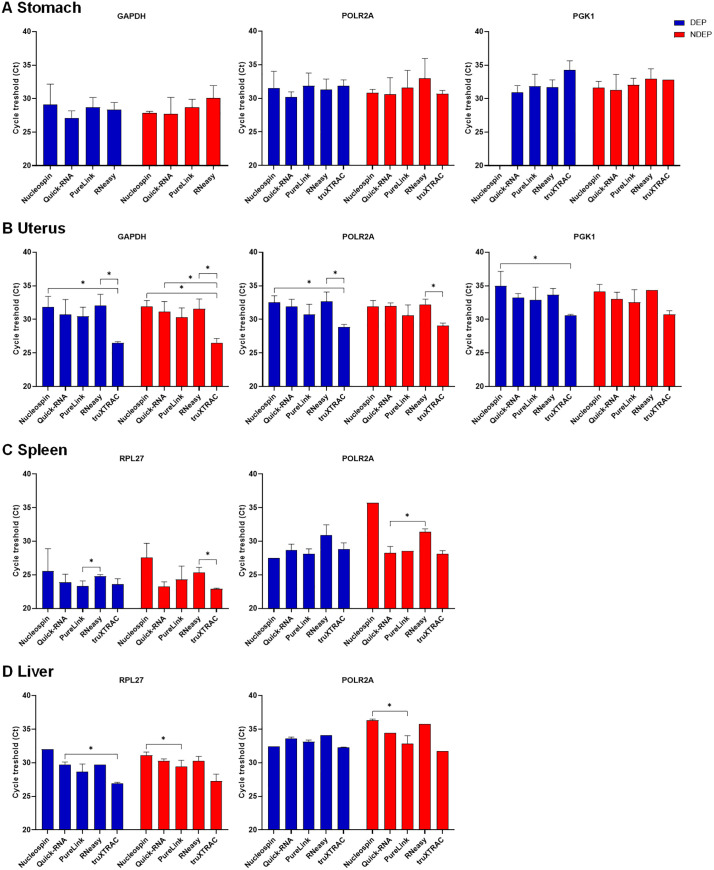
qPCR efficiency and target gene quantification among tested kits and conditions. RNAs extracted from deparaffinized (DEP) or non-deparaffinized (NDEP) microcores were analyzed by using each kit. Microcore analysis was performed from human stomach (A), human uterus (B), mouse spleen (C) or mouse liver (D). Samples were analyzed in duplicate. Duplicates with a standard deviation greater than 0.5 Ct are not shown here. The same amounts of cDNA were used for a single target gene. Kruskal Wallis with uncorrected Dunn’s test, *: p < 0.05.

Amplification and quantification of target genes confirmed that it was feasible to exploit extracted RNA from FFPE microcores using either the deparaffinized or non-deparaffinized protocols (Fig 6). Therefore, limitations associated with our microcore samples, specifically, the high level of RNA fragmentation and low amount of initial material resulted in a late amplification signal in a significant number of microcore samples, rendering their analysis infeasible. Consequently, some microcore samples are not represented here due to late and highly variable cycle threshold values.

Focusing on usable microcore samples, quantification of selected target genes by qPCR in RNA isolated from non-deparaffinized microcore samples was unaffected compared to deparaffinized microcore samples (Fig 6). No significant difference was observed between the two groups in all analyzed deparaffinized *vs* non-deparaffinized conditions demonstrating the applicability of this protocol to qPCR techniques.

Issues encountered when obtaining usable microcore samples limited statical analysis of our comparisons. Nevertheless, qPCR efficiency variability was observed depending on the kit used. The truXTRAC kit generated robust and reproducible results, particularly in the uterus (Fig 6B) and the liver (Fig 6D), with notable differences compared with the other kits. Together, with good reproducibility of results, these findings highlight the superiority of this kit for qPCR applications compared with alternative commercial kits. The Quick-RNA and PureLink kits also enabled the generation of exploitable and reproducible signals in the vast majority of the microcore samples analyzed (Fig 6). However, their efficiency depended on the tissue used.

The Nucleospin and RNeasy kits consistently generated less robust and reproducible results by giving late or undetectable Ct Values (Fig 6). This correlated with low yields obtained with these kits (Fig 1). Effect on performance is particularly noticeable in the uterus and liver when using the Nucleospin kit (Fig 6B,6D), and in the uterus and spleen when using the RNeasy kit (Fig 6B,6C). Therefore, constraints in microcore sample usability associated with the Nucleospin and RNeasy kits limited their applicability to this approach.

## Discussion

Routine diagnoses of pathologists are based on WHO histoprognostic classifications, sometimes with the help of molecular biology techniques. Isolation of a defined area within tissue sections or FFPE blocks enables pathologists to improve diagnostic accuracy. This is achieved by enhancing the signal associated with the population of interest and enabling access to intratumoral heterogeneity. The recent development of two microdissection platforms that allow rapid sampling rate (<1min/sample) of microcores from FFPE blocks is helping to make microdissection a more pertinent diagnostic tool that could be used routinely.

This study evaluates five commercial RNA extraction kits for their efficacy on 200 μm microcores from four tissue types, including a novel comparison of deparaffinized vs. non-deparaffinized protocols. We found that RNA yield and integrity varied significantly between kits, with the PureLink kit generally performing best. Crucially, omitting the deparaffinization step did not significantly impact RNA yield or qPCR efficiency, demonstrating a streamlined workflow for microcore analysis. RNA yields, which influence potential downstream applications, varied considerably between kits (Fig 1). For example, 10-fold differences in yields were observed between the kits in liver tissue (Table 2). The yields obtained were highly dependent on the tissue analyzed. Nevertheless, it is important to note that all conditions produced a concentration sufficient for molecular biology applications such as quantitative or droplet PCR where the technical limit is around 1 ng/µL (Table 2). It is well known that the average yield of samples composed predominantly of adipose tissue or with low cellularity would be lower compared to those observed in this study. In such cases, performing simultaneous extractions of several microcores samples originating from the same location may increase the final concentration and improve the usability. Limited microcore sample quantities can be a barrier for RNA-seq applications, as the substantial amount of information generated requires large input material. For FFPE samples, a threshold of 25 ng/µL is required to achieve adequate bioinformatic analyses [25]. As seen the concentrations obtained from a singular microcore can sometimes (depending on the tissue studied) below this threshold for RNAseq. To overcome this limitation several proximal microcores could be acquired to generate sufficient RNA for this application.

Pre-analytical variables such as the fixation process and storage conditions can significantly impact on the integrity of biological materials. To control for this bias, all microcore samples from a given tissue were derived from the same block. However, according to quality metrics, the post-extraction integrity of the RNA depended on the protocol used (Figs 2–5, Table 2). It was found that the RNeasy and truXTRAC kits caused increased RNA fragmentation compared to other kits. These differences may have an impact on downstream molecular applications, as Odogwu et al. reported that FFPE samples with a DV_200_ value above 30% can generate high-quality transcriptomic sequencing data [20]. To allow complete lysis of microcore samples, a 4 h proteinase K lysis time was set. Although RNAs are highly sensitive to temperature, lysis time did not result in significant RNA fragmentation which would prevent the use of sensitive downstream applications such as sequencing. The FFPE blocks used in this study were relatively recent (<3 months old) and stored under appropriate conditions (stable temperature and absence of humidity). As these factors are critical determinants of RNA integrity, it can be anticipated that less satisfactory results would likely have been obtained from older tissue blocks, especially those stored for more than 10 years or from those stored in less optimal conditions.

Amplification of the obtained RNA was optimized by designing short length primers. These “small” primers offset the side effects of strong RNA fragmentation (Table 1) and thus reduce the impact of fragmentation on qPCR reaction (Fig 6). The main limitation here was the small amounts of RNA available for each PCR reaction, which resulted in a late amplification signal and non-exploitable data. This issue was particularly evident with the Nucleospin and RNeasy kits.

The low RNA yield associated with the microcore samples meant that material losses had to be minimized during the extraction process. Furthermore, the small size of the microcore samples was associated with a risk of loss during handling. To mitigate this issue, we omitted the deparaffinization step, which is usually required for molecular analysis. In fact, as the cores were collected in tissue-rich areas, we confirmed that omitting this step had no significant impact on the RNA yield. Similar results were observed, for all tissues and kits used and with or without deparaffinization, demonstrating the applicability of this protocol. Although a slight increase in RNA degradation was observed under non-deparaffinized conditions for all kits, no significant impact on target gene quantification by qPCR was found.

Various studies have conducted similar comparisons, including those involving the extraction kits used in this study. Our findings are consistent with Patel *et al.,* who reported a similar hierarchy, with PureLink outperforming RNeasy and Nucleospin kits in extraction efficiency [16]. Their results further support our conclusions despite a different core size of 0.6 mm diameter was used in their study. Landolt *et al.* also reported increased RNA fragmentation when using the RNeasy kit on 5 µm rat whole kidney tissue sections, human kidney tissue sections and Laser Capture Microdissected glomerular cross-section [17]. Similar conclusions concerning the RNeasy kit were also made by Dube *et al.* on 20 µm thick tissue sections by comparing 9 commercially available kits. Suggesting an inherent characteristic of its chemistry. This study further highlights the PureLink kit efficiency, as well as that of Promega and Roche kits, not used in this study [18]. Nevertheless, a study conducted by Boeckx *et al.* on cervical cancer tissue sections highlighted the effectiveness of the RNeasy protocols compared to four other kits not studied examined in this study [19].

There are several limitations to our study. Firstly, while these results are reproducible across various biological tissues, the limited number of technical replicates means that the results should be interpreted with caution due to a restriction on statistical significance of our findings. Secondly, we chose to focus our study on the qPCR application, and this does not account for other molecular biology approaches, such as digital PCR and RNA-sequencing. As previously discussed, even if the quality of the extracted RNA is sufficient, obtaining an adequate signal with RNA-seq, using the microcores collected might prove challenging [25]. Nevertheless, new RNA-seq library preparation kits such as the Illumina’s RNA Prep with Enrichment (Illumina) have been developed, that allow the use of as little as 20 ng of FFPE-derived RNA. Using this new generation of libraries could enable the acquisition of adequate signals from RNA-seq using our microcores, however this remains the next major challenge. Furthermore, the choice of the most appropriate kit is crucial, as several studies have demonstrated that variations in FFPE RNA extraction protocols can impact both preanalytical and RNA sequencing results [21–23]. Lastly, as discussed above, we only included a limited number of FFPE RNA extraction kits, despite a wide range of protocols being available.

Despite challenges associated with generated microcores, we have shown that it is possible to extract sufficient RNA from 200 µm microscores for molecular biology analysis. However, this approach requires further validation using additional molecular techniques. Based on these findings, three of the five kits evaluated here, PureLink, Quick-RNA and truXTRAC, met our expectations and allowed efficient adaptation to the limitations inherent to FFPE microcores. Our work is the first to validate these protocols for 200 µm microcores. In this way, identification of reliable kits as well as the implementation of time saving protocols (non-deparaffinized) will provide an essential benchmark for laboratories adopting microcore technologies. Together with the recent commercialization of microcore-based microdissection techniques, this approach is the first to successfully integrate sampling accuracy with satisfactory throughput, while ensuring rational use of precious biological material. Expanding the use of microcore techniques and optimizing their application in both research and medical fields will enhance our understanding of the complex mechanisms linked with tumor progression and contribute to improved global patient care.

## Supporting information

S1 Fig Microcore collection and histological validation (A) Photomicrograph of a microcore collected from mouse liver (original magnification: X10). Bar scale: 200 µm. (B) Microcore localization on tissue section of mouse liver analysed by haematoxylin and eosin staining. Diameter of 400 µm (200 µm inner diameter/2*100 µm needle wall) corresponding to the sampling area. Bar scale: 500 µm. (C) Size distribution of microcores collected.
(PPTX)
