# Supplementary material for: Comparing RNA extraction protocols from formalin-fixed paraffin-embedded microcore samples
Source: PLoS One. 2025 Dec 8;20(12):e0338439. doi: 10.1371/journal.pone.0338439 (PMC12685168; doi:10.1371/journal.pone.0338439)
Supplement: S1 Fig — Bar scale: 200 µm. (B) Microcore localization on tissue section of mouse liver analysed by haematoxylin and eosin staining. Diameter of 400 µm (200 µm inner diameter/2*100 µm needle wall) corresponding to the sampling area. Bar scale: 500 µm. (C) Size distribution of microcores collected. (PPTX) [file pone.0338439.s001.pptx]

## Slide 1
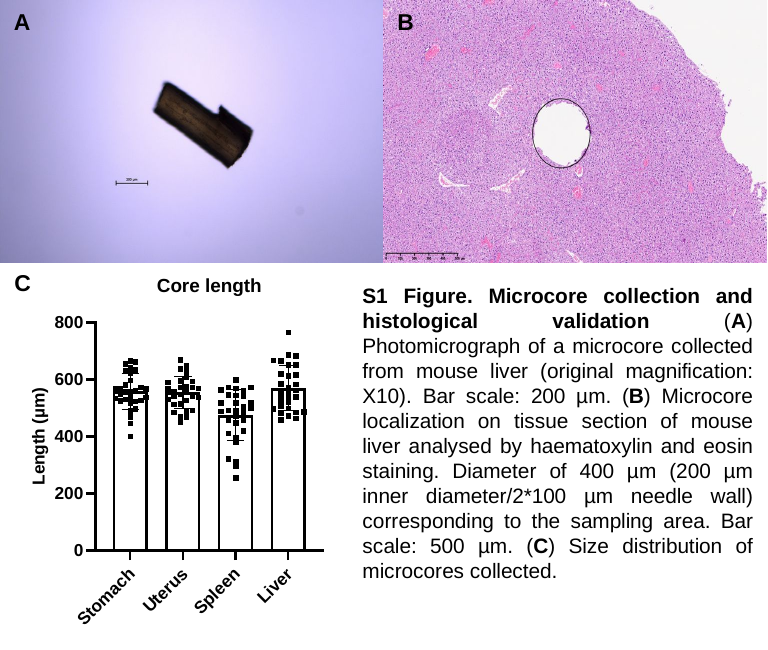

A
B
C
S1 Figure. Microcore collection and histological validation (A) Photomicrograph of a microcore collected from mouse liver (original magnification: X10). Bar scale: 200 µm. (B) Microcore localization on tissue section of mouse liver analysed by haematoxylin and eosin staining. Diameter of 400 µm (200 µm inner diameter/2*100 µm needle wall) corresponding to the sampling area. Bar scale: 500 µm. (C) Size distribution of microcores collected.
